# Crystal structures of *N*′-amino­pyridine-2-carboximidamide and *N*′-{[1-(pyridin-2-yl)ethyl­idene]amino}­pyridine-2-carboximidamide

**DOI:** 10.1107/S2056989017008416

**Published:** 2017-06-13

**Authors:** Francois Eya’ane Meva, Timothy John Prior, David John Evans, Emmanuel Roland Mang

**Affiliations:** a Department of Pharmaceutical Sciences, Faculty of Medicine and Pharmaceutical Sciences, University of Douala, PO Box 2701, Cameroon; b Chemistry, School of Mathematics and Physical Sciences, University of Hull, HU6 7RX, England; c Department of Chemistry, University of Douala, PO Box 24157, Cameroon

**Keywords:** crystal structure, pyridine-2-carboximidamide, *N*′-{[1-(pyridin-2-yl)ethyl­idene]amino}­pyridine-2-carboximidamide

## Abstract

The crystal structures of *N*′-amino­pyridine-2-carboximidamide (C_6_H_8_N_4_), **1**, and *N*′-{[1-(pyridin-2-yl)ethyl­idene]amino}­pyridine-2-carboximidamide (C_13_H_13_N_5_), **2**, are described. The non-H atoms in compound **1** are nearly planar (r.m.s. deviation from planarity = 0.0108 Å), while **2** is twisted about the central N—N bond by 17.8 (2)°. Both mol­ecules are linked by inter­molecular N—H⋯N hydrogen-bonding inter­actions; **1** forms a two-dimensional hydrogen-bonding network and for **2** the network is a one-dimensional chain. The bond lengths of these mol­ecules are similar to those in other literature reports of azine and di­imine systems.

## Abstract 2

In the crystal structures of *N*′-amino­pyridine-2-carboximidamide (C_6_H_8_N_4_), **1**, and *N*′-{[1-(pyridin-2-yl)ethyl­idene]amino}­pyridine-2-carboximidamide (C_13_H_13_N_5_), **2**, mol­ecules are linked by inter­molecular N—H⋯N hydrogen-bonding inter­actions, forming a two-dimensional network in **1** and a chain in **2**.

## Chemical context   

The preparation of hydrazidines with the general formula *R*C(=NH)NHNH_2_ is accomplished by the action of hydrazine on the corresponding thio­amide, imido ether or nitrile (Case, 1965 ▸). A pyridine-2-carboxamidrazide co-crystal form has previously been crystallized as a pyridine-2-carboxamidra­zonium hydrogenoxalate salt, obtained by the reaction of pyridine-2-carboxamidrazide with oxalic acid (Wang *et al.*, 2007 ▸). Related mol­ecules with diazine (N—N) bridges, obtained by condensation of hydrazidines with ketones can bring two metal centres into close proximity and provide an intra­molecular exchange pathway for spin-exchange inter­actions *via* the *p-*orbital system (σ pathway) of the heterocyclic ligand (Xu *et al.*, 1997 ▸, 2000 ▸). The latter type of mol­ecules present an unusual arrangement of potential donor sites, with many possible mononucleating and dinucleating coordination modes (Xu *et al.*, 1997 ▸). Semi-empirical structural calculations demonstrate that the N—N bond in these azines is rotationally soft, thereby allowing significant twisting at little energy cost (Kesslen *et al.*, 1999 ▸). Copper azine and imine complexes possess a significant anti­malarial and anti­tumor action (Gokhale *et al.*, 2001*a* ▸,*b* ▸, 2003 ▸). Coordination complexes of 2-acetyl­pyridine-pyridine-2-carboxamidrazone have been obtained with cadmium(II), copper(II), nickel(II) and manganese(II) ions. The organic mol­ecule behaves as a mono- and bis­(bidentate) chelator (Xu *et al.*, 2000 ▸; Gokhale *et al.*, 2001*a* ▸; Yue *et al.*, 2004 ▸, 2006 ▸). A polymorph of 2-acetyl­pyridine-pyridine-2-carboxamidrazone as been obtained with two crystallographically independent mol­ecules included in the asymmetric unit (Yue *et al.*, 2006 ▸).
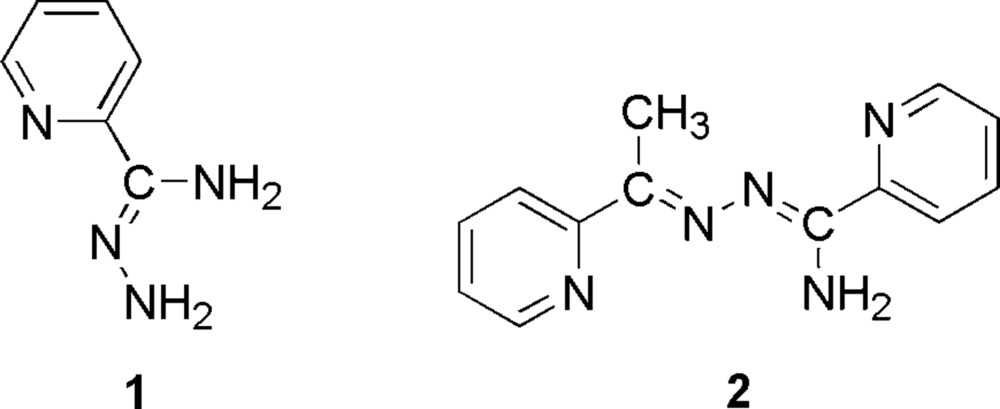


## Structural commentary   

**Figure 1:**
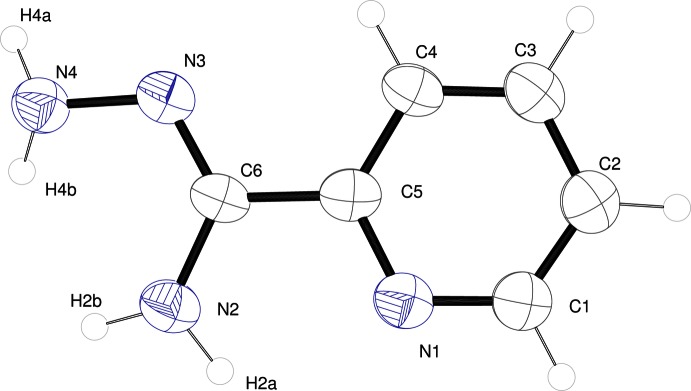
*ORTEP* representation of the asymmetric unit of **1**, with displacement ellipsoids drawn at the 50% probability level.

The mol­ecular structure of **1** is shown in Fig. 1 ▸. The mol­ecule is close to planar; the r.m.s. deviation of non-hydrogen atoms from planarity is 0.0108 Å with atom N2 displaying the largest deviation from the mean plane of 0.016 (3) Å. The geometry about N2 and N4 is not planar. H2*A* and H2*B* lie 0.12 (6) and 0.24 (6) Å out of the mean plane of non-hydrogen atoms. For H4*A* and H4*B*, the deviation is even greater at 0.37 (5) and 0.54 (5) Å from the mean plane. Rotation of the non-planar NH_2_ group, particularly for N4, facilitates hydrogen bonding to other mol­ecules. The N—N single bond length in **1** [1.424 (5) Å] is slightly shorter than that in the free hydrazine (1.449 Å).

**Figure 2:**
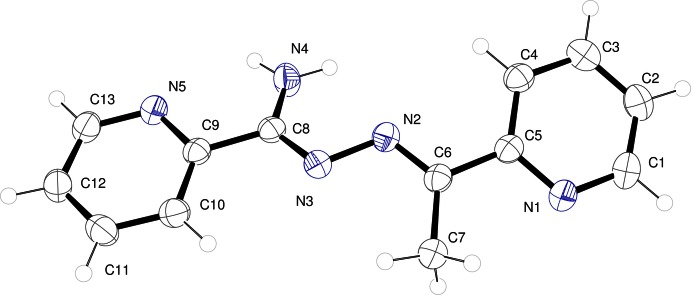
*ORTEP* representation of the asymmetric unit of **2**, with displacement ellipsoids drawn at the 50% probability level.

The mol­ecular structure of **2** is shown in Fig. 2 ▸. The mol­ecule is not planar, perhaps as a result of conjunction of supra­molecular inter­actions and packing effects. Each of the two ring systems is essentially planar (r.m.s. deviations for the two six-membered rings are 0.0162 and 0.0057 Å for N1/C1–C5 and N5/C9–C13, respectively). The hydrazidine group N3/C8/N4 is rotated slightly away from the plane of the six-membered ring along the C8—C9 bond by 8.6 (3)°. The imine group N2/C6/C7 is rotated from the plane of the adjacent six-membered ring by rotation about C5—C6 by 14.5 (2)°. The mol­ecule is further distorted away from planarity by rotation of 17.8 (2)° about the central N2—N3 bond.

The bond lengths indicate that within the central chain of the mol­ecule, the C6—N2 and C8—N3 linkages have largely double-bond character. The azine linkages are in the *E*,*E* conformation, suggesting conjugation throughout the π systems. The C6—N2—N3 and C8—N3—N2 angles of 115.5 (2)° and 110.57 (19)°, respectively are significantly below the ideal *sp* ^2^ value of 120°, a consequence of the repulsion between the nitro­gen lone pair and the adjacent bonds. The C6—N2—N3—C8 torsion angle is −162.2 (2)°. This large deviation from planarity has two consequences. First, there is a loss of conjugation between the imine bonds across the azine bond, reflected in the shorter imine bond length. The torsion also leads to a shorter N2—N3 bond length [1.408 (3) Å] compared to that observed for **1** [1.424 (5) Å]. Finally, a short intra­molecular contact between N3^i^ and H4*B*, 2.42 (3) Å, may add a favorable electrostatic contribution to the stability of this conformation. Notably, there is minimal change in the bond lengths within the ligands when a first row transition metal ion is bound. When the ligand chelates to a metal ion through both N3 and N5, only the bond length C8—N4 changes significantly, becoming shorter on binding.

## Supra­molecular features   

**Figure 3:**
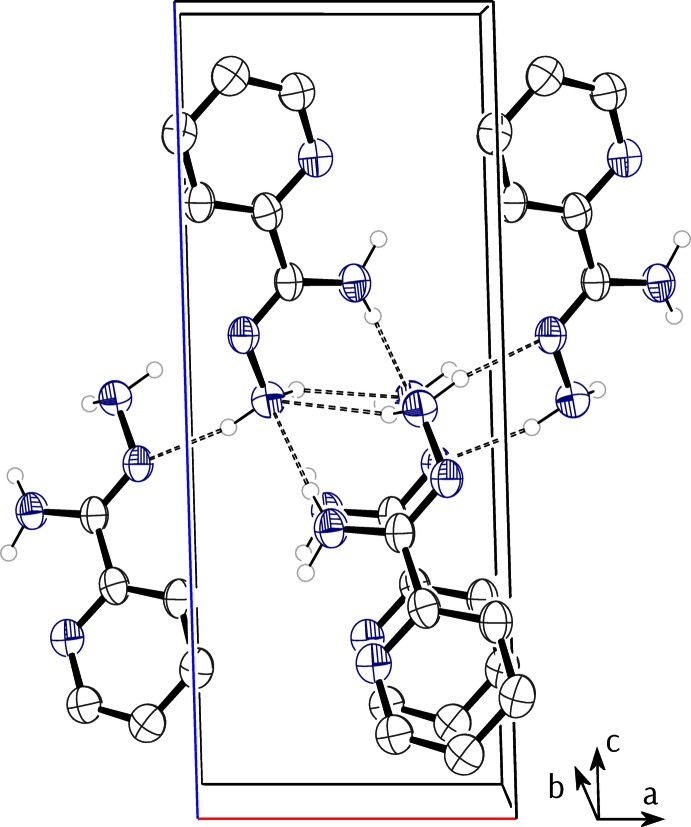
A portion of the hydrogen-bonded sheet present in **1**. Hydrogen bonds are shown as dashed lines.

There are two mol­ecules of **1** in each unit cell and these are related by the screw axis. Curiously, N1 does not act as a hydrogen-bond acceptor. H2*A* is also not involved with the formation of any (short) classical hydrogen bonds. H2*B* forms a hydrogen bond to N4^i^ [symmetry code: (i) 1 – *x*, *y* + 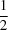, 1 – *z*]. This is augmented by the longer hydrogen bond N4—H4*B*⋯N4^i^. N4—H4*A* forms a hydrogen bond to N3^ii^ [symmetry code: (ii) –*x*, *y* + 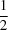, –*z* + 1]. These three sets of hydrogen bonds (Table 1 ▸) are sufficient to hold pairs of mol­ecules together within the unit cell and to knit these dimers together to form sheets in the *xy* plane (see Fig. 3 ▸). These sheets then stack parallel to the [001] direction, presumably held together by van der Waals inter­actions.

**Figure 4:**
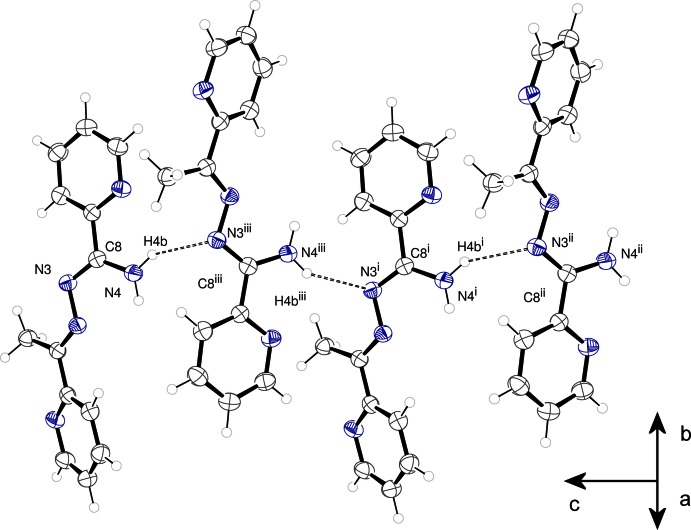
A portion of the hydrogen-bonded chain present in **2**. Hydrogen bonds are shown as dashed lines. Symmetry codes: (i) *x*, *y*, *z* − 1; (ii) 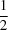 − *x*, 1 − *y*, *z* − 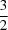; (iii) 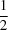 − *x*, 1 − *y*, *z* − 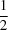.

The classical hydrogen bonding (Table 2 ▸) in **2** is more sparse than in **1**. N1, N2, and N5 do not act as classical hydrogen-bond acceptors. A single symmetry-independent hydrogen bond [N4—H4*B*⋯N3^i^ [symmetry code: (i) 1/2 – *x*, 1 – *y*, *z* – 1/2] is present and this knits the mol­ecules of **2** together to form hydrogen-bonded chains along the [001] direction, as shown in Fig. 4 ▸. There are subsidiary short C—H⋯N(pyridine) distances suggestive of inter­molecular inter­actions.

## Database survey   

For literature on *N*′-aminopyridine-2-carboximidamide and related mol­ecules, see Case *et al.* (1965 ▸). For the synthesis of *N*′-{[1-(pyridin-2-yl)ethylidene]amino}pyridine-2-carboximidamide and analogues, see Gokhale *et al.* (2001*a* ▸,*b* ▸, 2003 ▸) and Xu *et al.* (1997 ▸, 2000 ▸). For the coordination chemistry of *N*′-aminopyridine-2-carboximidamide, see Xu *et al.* (2000 ▸), Gokhale *et al.* (2001*a* ▸) and Yue *et al.* (2004 ▸, 2006 ▸).

## Synthesis and crystallization   

**Figure 5:**
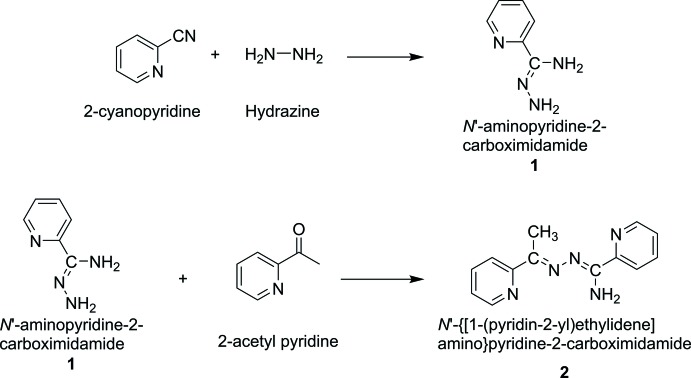
The synthesis of **1** and **2**.

The synthesis of *N*′-amino­pyridine-2-carboximidamide and *N*′-{[1-(pyridin-2-yl)ethyl­idene]amino}­pyridine-2-carboximid­amide is depicted in Fig. 5 ▸.

*N*′-Amino­pyridine-2-carboximidamide (**1**) was prepared by an analogy of the procedure published by Case (1965 ▸) with some modifications. A mixture of 2-cyano­pyridine (0.05 mol), absolute ethanol (9 ml), and 95% hydrazine (15 ml) was stirred at room temperature for 2 h. The solid product was then dried under vacuum and recrystallized from benzene. *N*′-{[1-(Pyridin-2-yl)ethyl­idene]amino}­pyridine-2-carboximid­amide (**2**) was synthesized by an analogy of the procedure published by Gokhale *et al.* (2001*a* ▸) by refluxing pyridine-2-carboxamidrazide (1) (0.5 g, 3.6 mmol) with excess 2-acetyl pyridine (0.5 g, 4.1 mmol) in absolute ethanol (20 ml) for 2 h. On cooling the product separates out in one week as yellow crystals which were filtered and dried.

## Refinement   

Crystal data, data collection and structure refinement details are summarized in Table 3 ▸.

There is no significant anomalous dispersion at this wavelength so the Flack parameter is meaningless and this is not reported.

For compound **1**, hydrogen atoms of the aromatic ring were placed using a riding model with the C—H bond length allowed to refine subject to the restraint that all these bond lengths were equal within a estimated standard deviation of 0.02 Å. These C—H bond lengths lie in the range 0.97 (3) to 0.99 (3) Å. The other hydrogen atoms attached to formally single-bonded nitro­gen atoms were freely refined subject to sensible distance and angle restraints. The N—H distances lie in the range 0.94 (3)-0.95 (3) Å.

For compound **2**, hydrogen atoms were placed using a riding model [N—H = 0.88, C—H = 0.95–0.98 Å; *U*_iso_(H) = 1.2 or 1.5*U*_eq_(C)].

## Supplementary Material

Crystal structure: contains datablock(s) 1, 2. DOI: 10.1107/S2056989017008416/nk2236sup1.cif

Structure factors: contains datablock(s) 1. DOI: 10.1107/S2056989017008416/nk22361sup2.hkl

Supporting information file. DOI: 10.1107/S2056989017008416/nk22361sup4.cdx

Structure factors: contains datablock(s) 2. DOI: 10.1107/S2056989017008416/nk22362sup3.hkl

Supporting information file. DOI: 10.1107/S2056989017008416/nk22362sup5.cdx

Supporting information file. DOI: 10.1107/S2056989017008416/nk22361sup6.cml

Supporting information file. DOI: 10.1107/S2056989017008416/nk22362sup7.cml

CCDC references: 1554569, 1554568

Additional supporting information:  crystallographic information; 3D view; checkCIF report

## Figures and Tables

**Table 1:** Hydrogen-bond geometry (Å, °) for **1** [Chem scheme1]

*D*—H⋯*A*	*D*—H	H⋯*A*	*D*⋯*A*	*D*—H⋯*A*
N2—H2*B*⋯N4^i^	0.95 (3)	2.16 (3)	3.106 (5)	174 (4)
N4—H4*B*⋯N4^i^	0.94 (3)	2.51 (3)	3.357 (6)	149 (4)
N4—H4*A*⋯N3^ii^	0.95 (3)	2.19 (4)	3.113 (5)	162 (4)

Symmetry codes: (i) 
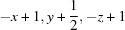
; (ii) 
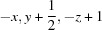
.

**Table 2:** Hydrogen-bond geometry (Å, °) for **2** [Chem scheme1]

*D*—H⋯*A*	*D*—H	H⋯*A*	*D*⋯*A*	*D*—H⋯*A*
N4—H4*B*⋯N3^i^	0.88	2.42	3.206 (3)	149

Symmetry code: (i) 
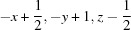
.

**Table 3:** Experimental details

	**1**	**2**
Crystal data		
Chemical formula	C_6_H_8_N_4_	C_13_H_13_N_5_
*M*_r_	136.16	239.28
Crystal system, space group	Monoclinic, *P*2_1_	Orthorhombic, *P*2_1_2_1_2_1_
Temperature (K)	150	150
*a*, *b*, *c* (Å)	5.6955 (14), 3.8408 (5), 14.592 (4)	6.6899 (5), 18.930 (2), 9.6561 (11)
α, β, γ (°)	90, 91.631 (19), 90	90, 90, 90
*V* (Å^3^)	319.08 (12)	1222.8 (2)
*Z*	2	4
Radiation type	Mo *K*α	Mo *K*α
μ (mm^−1^)	0.10	0.08
Crystal size (mm)	0.48 × 0.21 × 0.20	0.50 × 0.30 × 0.30

Data collection		
Diffractometer	Stoe IPDS2	Stoe IPDS2
Absorption correction	Multi-scan (*SORTAV*; Blessing, 1995 ▸)	–
*T*_min_, *T*_max_	0.909, 0.963	–
No. of measured, independent and observed [*I* > 2σ(*I*)] reflections	2271, 1407, 806	4734, 3172, 1881
*R*_int_	0.079	0.060
(sin θ/λ)_max_ (Å^−1^)	0.688	0.688

Refinement		
*R*[*F* ^2^ > 2σ(*F* ^2^)], *wR*(*F* ^2^), *S*	0.072, 0.193, 0.87	0.040, 0.085, 0.83
No. of reflections	1407	3172
No. of parameters	109	164
No. of restraints	12	0
H-atom treatment	H atoms treated by a mixture of independent and constrained refinement	H-atom parameters constrained
Δρ_max_, Δρ_min_ (e Å^−3^)	0.30, −0.37	0.13, −0.17

Computer programs: *X-AREA* (Stoe & Cie, 2005 ▸), *SORTAV* (Blessing, 1987 ▸, 1989 ▸), *SHELXT* (Sheldrick, 2015*a* ▸) and*SHELXL2014* (Sheldrick, 2015*b* ▸).

## supplementary crystallographic information

### (1) N'-Aminopyridine-2-carboximidamide Crystal data

**Table:**

C_6_H_8_N_4_	*F*(000) = 144
*M**_r_* = 136.16	*D*_x_ = 1.417 Mg m^−^^3^
Monoclinic, *P*2_1_	Mo *K*α radiation, λ = 0.71073 Å
*a* = 5.6955 (14) Å	Cell parameters from 2199 reflections
*b* = 3.8408 (5) Å	θ = 5.5–28.6°
*c* = 14.592 (4) Å	µ = 0.10 mm^−^^1^
β = 91.631 (19)°	*T* = 150 K
*V* = 319.08 (12) Å^3^	Block, colourless
*Z* = 2	0.48 × 0.21 × 0.20 mm

### (1) N'-Aminopyridine-2-carboximidamide Data collection

**Table:**

Stoe IPDS2 diffractometer	1407 independent reflections
Radiation source: fine-focus sealed tube	806 reflections with *I* > 2σ(*I*)
Detector resolution: 6.67 pixels mm^-1^	*R*_int_ = 0.079
ω–scan	θ_max_ = 29.3°, θ_min_ = 2.8°
Absorption correction: multi-scan (SORTAV; Blessing, 1995)	*h* = −7→6
*T*_min_ = 0.909, *T*_max_ = 0.963	*k* = −4→5
2271 measured reflections	*l* = −20→19

### (1) N'-Aminopyridine-2-carboximidamide Refinement

**Table:**

Refinement on *F*^2^	12 restraints
Least-squares matrix: full	Hydrogen site location: mixed
*R*[*F*^2^ > 2σ(*F*^2^)] = 0.072	H atoms treated by a mixture of independent and constrained refinement
*wR*(*F*^2^) = 0.193	*w* = 1/[σ^2^(*F*_o_^2^) + (0.1122*P*)^2^] where *P* = (*F*_o_^2^ + 2*F*_c_^2^)/3
*S* = 0.87	(Δ/σ)_max_ < 0.001
1407 reflections	Δρ_max_ = 0.30 e Å^−^^3^
109 parameters	Δρ_min_ = −0.37 e Å^−^^3^

### (1) N'-Aminopyridine-2-carboximidamide Special details

**Table:**

Geometry. All esds (except the esd in the dihedral angle between two l.s. planes) are estimated using the full covariance matrix. The cell esds are taken into account individually in the estimation of esds in distances, angles and torsion angles; correlations between esds in cell parameters are only used when they are defined by crystal symmetry. An approximate (isotropic) treatment of cell esds is used for estimating esds involving l.s. planes.

### (1) N'-Aminopyridine-2-carboximidamide Fractional atomic coordinates and isotropic or equivalent isotropic displacement parameters (Å^2^)

**Table:**

	*x*	*y*	*z*	*U*_iso_*/*U*_eq_	
C1	0.3776 (8)	0.3892 (13)	0.8928 (3)	0.0457 (11)	
H1	0.494 (4)	0.4064 (14)	0.9427 (17)	0.055*	
C2	0.1680 (8)	0.2331 (13)	0.9109 (3)	0.0464 (11)	
H2	0.1358 (13)	0.143 (3)	0.973 (2)	0.056*	
C3	0.0007 (9)	0.2040 (13)	0.8396 (3)	0.0462 (11)	
H3	−0.152 (5)	0.092 (4)	0.8496 (4)	0.055*	
C4	0.0545 (8)	0.3357 (12)	0.7545 (3)	0.0423 (11)	
H4	−0.060 (4)	0.3176 (14)	0.7027 (17)	0.051*	
C5	0.2711 (7)	0.4937 (12)	0.7426 (3)	0.0391 (9)	
C6	0.3370 (7)	0.6479 (12)	0.6539 (3)	0.0369 (9)	
N1	0.4337 (6)	0.5206 (10)	0.8109 (2)	0.0435 (10)	
N2	0.5525 (7)	0.7974 (11)	0.6517 (2)	0.0443 (10)	
N3	0.1844 (6)	0.6252 (9)	0.5862 (2)	0.0389 (9)	
N4	0.2585 (6)	0.7713 (12)	0.5022 (2)	0.0422 (9)	
H4A	0.125 (7)	0.840 (13)	0.466 (3)	0.063*	
H2A	0.637 (8)	0.830 (16)	0.708 (2)	0.063*	
H2B	0.614 (7)	0.954 (13)	0.608 (2)	0.063*	
H4B	0.353 (7)	0.970 (12)	0.512 (3)	0.065*	

### (1) N'-Aminopyridine-2-carboximidamide Atomic displacement parameters (Å^2^)

**Table:**

	*U*^11^	*U*^22^	*U*^33^	*U*^12^	*U*^13^	*U*^23^
C1	0.046 (3)	0.041 (3)	0.050 (2)	0.002 (2)	0.0008 (18)	0.000 (2)
C2	0.050 (3)	0.036 (3)	0.053 (2)	0.006 (2)	0.0041 (19)	0.0020 (19)
C3	0.042 (2)	0.037 (3)	0.060 (2)	0.003 (2)	0.0060 (18)	0.0037 (18)
C4	0.035 (2)	0.035 (3)	0.056 (2)	−0.001 (2)	0.0017 (18)	−0.0012 (19)
C5	0.035 (2)	0.031 (2)	0.051 (2)	0.007 (2)	−0.0021 (16)	−0.0032 (17)
C6	0.030 (2)	0.029 (2)	0.052 (2)	0.0006 (19)	0.0007 (16)	−0.0010 (17)
N1	0.041 (2)	0.037 (2)	0.0518 (19)	0.0003 (19)	0.0005 (14)	0.0001 (16)
N2	0.039 (2)	0.041 (2)	0.0528 (19)	−0.0051 (19)	0.0004 (15)	0.0007 (17)
N3	0.0346 (18)	0.033 (2)	0.0486 (18)	0.0019 (17)	0.0014 (14)	−0.0004 (16)
N4	0.041 (2)	0.038 (2)	0.0469 (17)	−0.0008 (19)	0.0006 (14)	0.0039 (16)

### (1) N'-Aminopyridine-2-carboximidamide Geometric parameters (Å, º)

**Table:**

C1—N1	1.345 (6)	C5—N1	1.345 (5)
C1—C2	1.368 (7)	C5—C6	1.481 (5)
C1—H1	0.97 (3)	C6—N3	1.300 (5)
C2—C3	1.395 (7)	C6—N2	1.357 (6)
C2—H2	0.99 (3)	N2—H2A	0.95 (3)
C3—C4	1.383 (6)	N2—H2B	0.95 (3)
C3—H3	0.98 (3)	N3—N4	1.424 (5)
C4—C5	1.390 (6)	N4—H4A	0.95 (3)
C4—H4	0.99 (3)	N4—H4B	0.94 (3)
			
N1—C1—C2	124.5 (4)	N1—C5—C6	115.5 (4)
N1—C1—H1	117.8	C4—C5—C6	122.1 (4)
C2—C1—H1	117.8	N3—C6—N2	126.6 (4)
C1—C2—C3	118.2 (4)	N3—C6—C5	117.2 (4)
C1—C2—H2	120.9	N2—C6—C5	116.2 (3)
C3—C2—H2	120.9	C1—N1—C5	117.0 (4)
C4—C3—C2	118.4 (4)	C6—N2—H2A	118 (3)
C4—C3—H3	120.8	C6—N2—H2B	129 (2)
C2—C3—H3	120.8	H2A—N2—H2B	108 (3)
C3—C4—C5	119.5 (4)	C6—N3—N4	114.8 (4)
C3—C4—H4	120.3	N3—N4—H4A	110 (3)
C5—C4—H4	120.3	N3—N4—H4B	111 (3)
N1—C5—C4	122.4 (4)	H4A—N4—H4B	108 (3)

### (1) N'-Aminopyridine-2-carboximidamide Hydrogen-bond geometry (Å, º)

**Table:**

*D*—H···*A*	*D*—H	H···*A*	*D*···*A*	*D*—H···*A*
N2—H2*B*···N4^i^	0.95 (3)	2.16 (3)	3.106 (5)	174 (4)
N4—H4*B*···N4^i^	0.94 (3)	2.51 (3)	3.357 (6)	149 (4)
N4—H4*A*···N3^ii^	0.95 (3)	2.19 (4)	3.113 (5)	162 (4)

Symmetry codes: (i) −*x*+1, *y*+1/2, −*z*+1; (ii) −*x*, *y*+1/2, −*z*+1.

### (2) N'-{[1-(Pyridin-2-yl)ethylidene]amino}pyridine-2-carboximidamide Crystal data

**Table:**

C_13_H_13_N_5_	*D*_x_ = 1.300 Mg m^−^^3^
*M**_r_* = 239.28	Mo *K*α radiation, λ = 0.71073 Å
Orthorhombic, *P*2_1_2_1_2_1_	Cell parameters from 3070 reflections
*a* = 6.6899 (5) Å	θ = 2.2–27.9°
*b* = 18.930 (2) Å	µ = 0.08 mm^−^^1^
*c* = 9.6561 (11) Å	*T* = 150 K
*V* = 1222.8 (2) Å^3^	Block, yellow
*Z* = 4	0.50 × 0.30 × 0.30 mm
*F*(000) = 504	

### (2) N'-{[1-(Pyridin-2-yl)ethylidene]amino}pyridine-2-carboximidamide Data collection

**Table:**

Stoe IPDS2 diffractometer	1881 reflections with *I* > 2σ(*I*)
Radiation source: fine-focus sealed tube	*R*_int_ = 0.060
Detector resolution: 6.67 pixels mm^-1^	θ_max_ = 29.3°, θ_min_ = 2.2°
ω–scan	*h* = −7→9
4734 measured reflections	*k* = −22→25
3172 independent reflections	*l* = −13→9

### (2) N'-{[1-(Pyridin-2-yl)ethylidene]amino}pyridine-2-carboximidamide Refinement

**Table:**

Refinement on *F*^2^	0 restraints
Least-squares matrix: full	Hydrogen site location: inferred from neighbouring sites
*R*[*F*^2^ > 2σ(*F*^2^)] = 0.040	H-atom parameters constrained
*wR*(*F*^2^) = 0.085	*w* = 1/[σ^2^(*F*_o_^2^) + (0.0355*P*)^2^] where *P* = (*F*_o_^2^ + 2*F*_c_^2^)/3
*S* = 0.83	(Δ/σ)_max_ < 0.001
3172 reflections	Δρ_max_ = 0.13 e Å^−^^3^
164 parameters	Δρ_min_ = −0.17 e Å^−^^3^

### (2) N'-{[1-(Pyridin-2-yl)ethylidene]amino}pyridine-2-carboximidamide Special details

**Table:**

Geometry. All esds (except the esd in the dihedral angle between two l.s. planes) are estimated using the full covariance matrix. The cell esds are taken into account individually in the estimation of esds in distances, angles and torsion angles; correlations between esds in cell parameters are only used when they are defined by crystal symmetry. An approximate (isotropic) treatment of cell esds is used for estimating esds involving l.s. planes.

### (2) N'-{[1-(Pyridin-2-yl)ethylidene]amino}pyridine-2-carboximidamide Fractional atomic coordinates and isotropic or equivalent isotropic displacement parameters (Å^2^)

**Table:**

	*x*	*y*	*z*	*U*_iso_*/*U*_eq_	
C1	0.8155 (4)	0.19604 (14)	0.7310 (3)	0.0423 (6)	
H1	0.8311	0.1496	0.7666	0.051*	
C2	0.9604 (4)	0.22137 (15)	0.6439 (3)	0.0410 (6)	
H2	1.0716	0.1929	0.6189	0.049*	
C3	0.9409 (4)	0.28972 (15)	0.5928 (3)	0.0399 (6)	
H3	1.0393	0.3092	0.5331	0.048*	
C4	0.7756 (4)	0.32843 (13)	0.6309 (3)	0.0338 (6)	
H4	0.7596	0.3754	0.5981	0.041*	
C5	0.6314 (4)	0.29863 (12)	0.7179 (2)	0.0294 (5)	
C6	0.4460 (4)	0.33685 (11)	0.7576 (2)	0.0292 (5)	
C7	0.3185 (4)	0.30756 (13)	0.8722 (3)	0.0352 (6)	
H7A	0.2303	0.3447	0.9076	0.053*	
H7B	0.4045	0.2904	0.9472	0.053*	
H7C	0.2377	0.2684	0.8365	0.053*	
C8	0.1767 (4)	0.47107 (12)	0.6220 (3)	0.0289 (5)	
C9	−0.0076 (4)	0.51400 (12)	0.6439 (2)	0.0287 (5)	
C10	−0.1320 (4)	0.50366 (13)	0.7570 (3)	0.0350 (6)	
H10	−0.1022	0.4685	0.8240	0.042*	
C11	−0.3006 (4)	0.54571 (14)	0.7701 (3)	0.0405 (6)	
H11	−0.3877	0.5401	0.8470	0.049*	
C12	−0.3401 (4)	0.59572 (14)	0.6701 (3)	0.0390 (6)	
H12	−0.4544	0.6253	0.6767	0.047*	
C13	−0.2093 (4)	0.60183 (14)	0.5600 (3)	0.0382 (6)	
H13	−0.2371	0.6365	0.4915	0.046*	
N1	0.6516 (3)	0.23293 (11)	0.7696 (2)	0.0361 (5)	
N2	0.4076 (3)	0.39235 (11)	0.6850 (2)	0.0312 (5)	
N3	0.2326 (3)	0.42907 (10)	0.7220 (2)	0.0312 (5)	
N4	0.2661 (3)	0.47642 (11)	0.4976 (2)	0.0396 (5)	
H4A	0.3712	0.4503	0.4786	0.048*	
H4B	0.2193	0.5061	0.4354	0.048*	
N5	−0.0458 (3)	0.56182 (11)	0.5440 (2)	0.0344 (5)	

### (2) N'-{[1-(Pyridin-2-yl)ethylidene]amino}pyridine-2-carboximidamide Atomic displacement parameters (Å^2^)

**Table:**

	*U*^11^	*U*^22^	*U*^33^	*U*^12^	*U*^13^	*U*^23^
C1	0.0446 (16)	0.0378 (13)	0.0444 (15)	0.0104 (13)	−0.0050 (13)	0.0018 (12)
C2	0.0358 (15)	0.0472 (15)	0.0399 (15)	0.0085 (13)	0.0000 (13)	−0.0054 (13)
C3	0.0345 (15)	0.0476 (16)	0.0376 (15)	−0.0026 (13)	0.0011 (12)	−0.0038 (12)
C4	0.0354 (14)	0.0309 (13)	0.0349 (14)	−0.0025 (11)	−0.0012 (12)	−0.0004 (11)
C5	0.0322 (13)	0.0266 (12)	0.0295 (12)	−0.0028 (11)	−0.0045 (11)	−0.0015 (10)
C6	0.0309 (13)	0.0251 (11)	0.0315 (13)	−0.0016 (10)	−0.0029 (11)	−0.0024 (10)
C7	0.0347 (14)	0.0327 (13)	0.0382 (14)	0.0020 (12)	0.0027 (12)	0.0026 (11)
C8	0.0290 (12)	0.0251 (12)	0.0326 (13)	−0.0034 (10)	0.0001 (11)	−0.0021 (10)
C9	0.0293 (12)	0.0258 (11)	0.0310 (12)	−0.0028 (10)	−0.0023 (10)	−0.0008 (10)
C10	0.0350 (13)	0.0334 (13)	0.0368 (14)	−0.0022 (11)	0.0006 (12)	0.0019 (11)
C11	0.0336 (14)	0.0454 (15)	0.0424 (14)	−0.0003 (12)	0.0072 (13)	−0.0018 (12)
C12	0.0354 (15)	0.0344 (14)	0.0472 (16)	0.0053 (12)	0.0009 (13)	−0.0013 (12)
C13	0.0397 (15)	0.0313 (13)	0.0437 (15)	0.0032 (13)	−0.0015 (12)	0.0036 (12)
N1	0.0361 (12)	0.0324 (11)	0.0398 (12)	0.0037 (10)	−0.0019 (10)	0.0045 (9)
N2	0.0327 (11)	0.0281 (11)	0.0326 (11)	0.0016 (10)	−0.0001 (9)	−0.0009 (9)
N3	0.0296 (11)	0.0301 (11)	0.0341 (11)	0.0013 (9)	0.0028 (9)	0.0009 (9)
N4	0.0389 (13)	0.0462 (13)	0.0338 (11)	0.0138 (11)	0.0048 (10)	0.0074 (10)
N5	0.0340 (12)	0.0299 (11)	0.0392 (12)	0.0025 (10)	−0.0003 (10)	0.0023 (9)

### (2) N'-{[1-(Pyridin-2-yl)ethylidene]amino}pyridine-2-carboximidamide Geometric parameters (Å, º)

**Table:**

C1—N1	1.352 (3)	C8—N3	1.305 (3)
C1—C2	1.371 (4)	C8—N4	1.346 (3)
C1—H1	0.9500	C8—C9	1.492 (3)
C2—C3	1.391 (4)	C9—N5	1.347 (3)
C2—H2	0.9500	C9—C10	1.386 (4)
C3—C4	1.377 (4)	C10—C11	1.387 (3)
C3—H3	0.9500	C10—H10	0.9500
C4—C5	1.398 (3)	C11—C12	1.378 (4)
C4—H4	0.9500	C11—H11	0.9500
C5—N1	1.347 (3)	C12—C13	1.381 (4)
C5—C6	1.487 (3)	C12—H12	0.9500
C6—N2	1.289 (3)	C13—N5	1.340 (3)
C6—C7	1.503 (3)	C13—H13	0.9500
C7—H7A	0.9800	N2—N3	1.408 (3)
C7—H7B	0.9800	N4—H4A	0.8800
C7—H7C	0.9800	N4—H4B	0.8800
			
N1—C1—C2	124.2 (3)	N3—C8—C9	117.6 (2)
N1—C1—H1	117.9	N4—C8—C9	116.9 (2)
C2—C1—H1	117.9	N5—C9—C10	123.0 (2)
C1—C2—C3	118.5 (3)	N5—C9—C8	114.9 (2)
C1—C2—H2	120.8	C10—C9—C8	122.0 (2)
C3—C2—H2	120.8	C9—C10—C11	118.6 (2)
C4—C3—C2	118.4 (3)	C9—C10—H10	120.7
C4—C3—H3	120.8	C11—C10—H10	120.7
C2—C3—H3	120.8	C12—C11—C10	119.1 (3)
C3—C4—C5	120.0 (2)	C12—C11—H11	120.5
C3—C4—H4	120.0	C10—C11—H11	120.5
C5—C4—H4	120.0	C11—C12—C13	118.4 (3)
N1—C5—C4	121.7 (2)	C11—C12—H12	120.8
N1—C5—C6	116.0 (2)	C13—C12—H12	120.8
C4—C5—C6	122.3 (2)	N5—C13—C12	124.0 (2)
N2—C6—C5	115.0 (2)	N5—C13—H13	118.0
N2—C6—C7	126.0 (2)	C12—C13—H13	118.0
C5—C6—C7	118.9 (2)	C5—N1—C1	117.2 (2)
C6—C7—H7A	109.5	C6—N2—N3	115.5 (2)
C6—C7—H7B	109.5	C8—N3—N2	110.57 (19)
H7A—C7—H7B	109.5	C8—N4—H4A	120.0
C6—C7—H7C	109.5	C8—N4—H4B	120.0
H7A—C7—H7C	109.5	H4A—N4—H4B	120.0
H7B—C7—H7C	109.5	C13—N5—C9	116.9 (2)
N3—C8—N4	125.4 (2)		

### (2) N'-{[1-(Pyridin-2-yl)ethylidene]amino}pyridine-2-carboximidamide Hydrogen-bond geometry (Å, º)

**Table:**

*D*—H···*A*	*D*—H	H···*A*	*D*···*A*	*D*—H···*A*
N4—H4*B*···N3^i^	0.88	2.42	3.206 (3)	149

Symmetry code: (i) −*x*+1/2, −*y*+1, *z*−1/2.
